# Activation of Cryptic Antibiotic Biosynthetic Gene Clusters Guided by RNA-seq Data from Both *Streptomyces ansochromogenes* and ΔwblA

**DOI:** 10.3390/antibiotics10091097

**Published:** 2021-09-10

**Authors:** Yue Li, Haiying Yu, Hanye Guan, Jingjing Li, Jihui Zhang, Hua Xiang, Jine Li, Huarong Tan

**Affiliations:** 1 State Key Laboratory of Microbial Resources, Institute of Microbiology, Chinese Academy of Sciences, Beijing 100101, China; liyue@im.ac.cn (Y.L.); yuhaiying@im.ac.cn (H.Y.); hyguan@126.com (H.G.); jjingli@sina.com (J.L.); zhang.jihui@im.ac.cn (J.Z.); xiangh@im.ac.cn (H.X.); 2 College of Life Sciences, University of Chinese Academy of Sciences, Beijing 100049, China

**Keywords:** RNA-seq analysis, *Streptomyces*, cryptic gene cluster, tylosin analogue compounds 1,2,3 (TACs), oviedomycin

## Abstract

With the increase of drug resistance caused by the improper use and abuse of antibiotics, human beings are facing a global health crisis. Sequencing of *Streptomyces* genomes revealed the presence of an important reservoir of secondary metabolic gene clusters for previously unsuspected products with potentially valuable bioactivity. It has therefore become necessary to activate these cryptic pathways through various strategies. Here, we used RNA-seq data to perform a comparative transcriptome analysis of *Streptomyces ansochromogenes* (wild-type, WT) and its global regulatory gene disruption mutant ΔwblA, in which some differentially expressed genes are associated with the abolished nikkomycin biosynthesis and activated tylosin analogue compounds (TACs) production, and also with the oviedomycin production that is induced by the genetic manipulation of two differentially expressed genes (*san7324* and *san7324L*) encoding RsbR. These results provide a significant clue for the discovery of new drug candidates and the activation of cryptic biosynthetic gene clusters.

## 1. Introduction

Natural products from microbes play an important role in clinical treatments, animal husbandry and plant crop protection [1,2]. Actinomycetes, especially streptomycetes, are a particularly abundant source of secondary metabolites. The decreased number in clinical antibiotics and the emergence of antibiotic resistance pose new challenges for the discovery of novel antibiotics. Streptomycetes have the potential to produce more new natural products than those previously recognized under laboratory conditions. With the rapid development of genome sequencing technology and analysis tools, more and more cryptic secondary metabolites have been mined and the activated cryptic gene clusters have been identified through various strategies, which provide more opportunities to discover new natural products or drug candidates [3,4].

Currently used or proposed methods for activating the ‘cryptic’ pathways include the optimization of culture and fermentation conditions, heterologous expression, genetic manipulations of regulatory genes or gene clusters, omic studies, co-culture of different microbes, induction of signaling molecules, ribosomal engineering, reporter-guided strategy, and so on [5,6,7]. Meanwhile, microbial natural product databases, such as ’The Natural Products Atlas’ (https://www.npatlas.org/, accessed on 9 September 2021), antiSMASH database (https://antismash.secondarymetabolites.org, accessed on 9 September 2021), and CH-NMR-NP database (https://www.j-resonance.com/en/nmrdb/, accessed on 9 September 2021) play a key action in the mining of drug candidates [8,9,10]. Various bioinformatics tools have been developed and widely used in genomics or transcriptomics, such as Canu [11], Circos [12], RSEM (http://deweylab.github.io/RSEM/, accessed on 9 September 2021) [13], edgeR (http://www.bioconductor.org/packages/2.12/bioc/html/edgeR.html, accessed on 9 September 2021) [14] and DEGs KEGG enrichment analysis (KOBAS 2.0, http://kobas.cbi.pku.edu.cn, accessed on 9 September 2021) [15].

There are many successful examples for mining of hidden natural products. For instance, (i) Three natural products, streptothricin, geosmin and strevertene A (polyene), were successfully activated by using the reporter-guided strategy [16]; (ii) Multiplex activation strategies were implemented to awaken a cryptic biosynthetic gene cluster, resulting in the discovery of eight aromatic polyketides with two types of frameworks, two pentacyclic isomers and six glycosylated tetracyclines [17]; (iii) In *Streptomyces roseosporus*, mureidomycin biosynthesis was activated by the introduction of an exogenous regulatory gene *ssaA* [18].

The WhiB-like (Wbl) family of proteins broadly distribute in *Actinobacteria*, such as *Streptomyces, Corynebacteria* and *Mycobacteria* [19]. Wbls usually play key roles in the differentiation, the biosynthesis of various secondary metabolites, and the involvement in the oxidative stress response [19,20,21]. For instance, *wblA* plays a crucial role in the repression of daptomycin biosynthesis, which is probably mediated by the regulatory genes *atrA*, *dptR2* and *dptR3*. Although WblA has been reported to have a DNA-binding HTH domain, there are few examples of direct evidence about its transcriptional regulation from EMSA experiments [22]. Wbl proteins may change their regulatory function in response to O_2_ and nitric oxidation (NO) via iron-sulphur clusters essential for nutrient starvation survival [23]. In addition, some Wbls can interact with their partner proteins to modulate antibiotic resistance [24,25]. *Streptomyces ansochromogenes* 7100 was isolated from the northeast soil of China [26]. It can produce a peptidyl nucleoside compound nikkomycin, a promising antibiotic against phytopathogenic fungi and human pathogens [27]. Our previous work showed that the biosynthesis of novel cryptic tylosin analogue compounds 1,2,3 (TAC1,2,3 or TACs) with much higher activity than tylosin against *Streptococcus pneumonia* was activated by the disruption of *wblA*, meanwhile, nikkomycin was abolished [28].

In this study, we describe the comparative transcriptome analysis, in which some differentially expressed genes are related to the gene cluster’s activation/inactivation. This would provide an efficient approach for the activation of secondary metabolites guided by RNA-seq data.

## 2. Results and Discussion

### 2.1. Sequencing and AntiSMASH Analysis of S. Ansochromogenes 7100 Genome

In order to obtain a complete genome sequence of *S. ansochromogenes* 7100, the third generation sequencing was performed on a PacBio RSII platform. A total of 157,549 quality subreads were obtained in genome sequencing with an approximate coverage fold of more than 100× (Table S1, Supplementary Materials).

**Figure 1:**
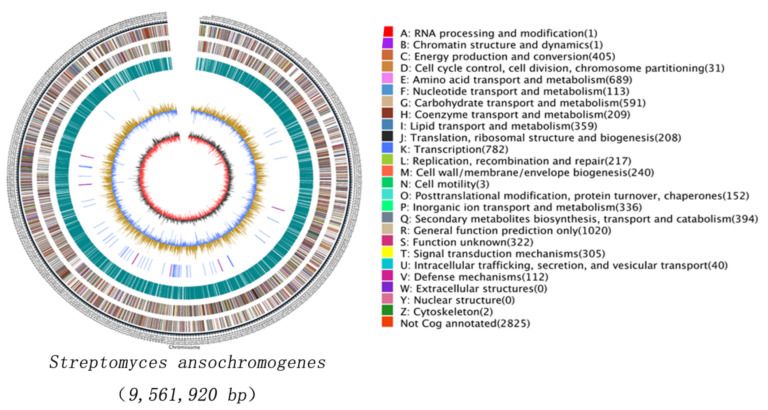
Circular representation of the *Streptomyces ansochromogenes* chromosome. The circle 1 (outer circle) is a marker of genome total size with a scale of 5 kb each; circles 2 and 3 (from the outside in) are genes on the forward and reverse strand respectively, which are color-coded by function; circle 4 indicates the distributed position of repeat sequence; circle 5 represents tRNAs and rRNAs; circle 6 shows GC content (the light yellow part indicates that the GC content in this region is higher than the average GC content in the genome, the blue part indicates that the GC content in this region is lower than the average GC content in the genome), circle 7 (inner circle) is GC-skew (Black represents the area where G content is greater than C, and red represents the area where C content is greater than G).

Then the raw data were filtered and assembled into a nearly complete genome of 9.56 Mb (Table S2) with software Canu. The main features of the *S. ansochromogenes* 7100 chromosome are shown in Figure 1. The genome of *S. ansochromogenes* 7100 is a linear chromosome with a repetitive sequence content of 2.37% (Table S3), which is composed of 8235 protein-coding genes (Table S4), 72 tRNAs, 18 rRNA and 50 nc RNAs genes (Table S5). Of the total gene set, 98% were classified into functional categories according to NR, COG, Pfam, KEGG pathway and Gene Ontology database (Table S6). The average G + C content of the genome is 72.41%.

The potential secondary metabolites biosynthetic gene clusters in *S. ansochromogenes* 7100 were analyzed by the antiSMASH program [10,29]. A total of 41 gene clusters were identified, which include nikkomycin gene cluster, TACs gene cluster and 39 uncharacterized gene clusters that are predicted to be involved in the biosynthesis of polyketides (PKSs), lassopeptide, terpene, lanthipeptide, bacteriocin, non-ribosomal peptides (NRPSs), terpene, butyrolactone, betalactone, siderophore, ectoine, melanin and so on (Table 1). Although only a few secondary metabolites have been identified, the abundant ‘cryptic’ gene clusters in *S. ansochromogenes* 7100 implied the potential for discovery of novel bioactive products.

### 2.2. Genome-Wide Differentially Expressed Genes in ΔwblA

*wblA* is an important regulatory gene usually involved in the sporulation, biosynthesis of secondary metabolites and oxidative stress in *Streptomyces* [21]. The disruption of *wblA* in *S. ansochromogenes* 7100 caused the failure of sporulation and the loss of nikkomycins production [28]. Instead, three novel 16-membered tylosin-like macrolides were accumulated in the resulting mutant strain (ΔwblA), arousing much interest in how the regulatory pathway works.

To elucidate the landscape of transcriptomic changes of *S. ansochromogenes* 7100 and ΔwblA, RNA-seq transcriptional profiles at 24 h and 48 h were analyzed. The production of TACs was not detectable at 24 h but appeared at 48 h with the subsequent expression of the biosynthetic genes. RNAs were isolated from the WT and ΔwblA strains at the two time points for cDNA synthesis, library construction and sequencing. The transcriptome sequencing of four libraries resulted in a total unique map of 76,486,734 sequence reads, which were aligned to the reference genome of *S. ansochromogenes* 7100 (Table S7). More than 95% of unique mapped reads ratio in each library mapped to the *S. ansochromogenes* genome. The data reached saturation (Figure S1).

**Figure 2:**
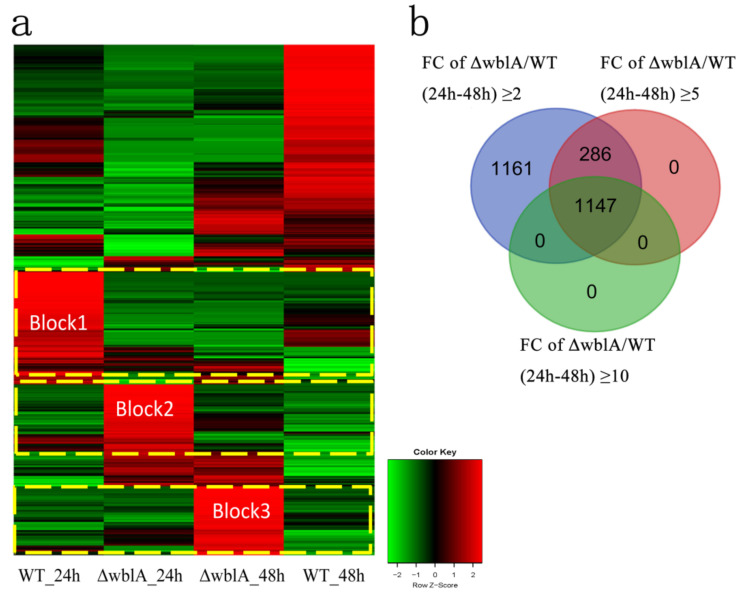
Heatmap and Venn diagram of gene transcriptional profiles. (**a**) Heatmap represents all differentially expressed genes whose transcriptional change ≥ 2–fold at 24 h and 48 h in both WT and ΔwblA strains, respectively. *p*_adjust_ < 0.001. Red and green colors represent up-regulated and down-regulated genes, respectively. (**b**) The Venn diagram represents the numbers of differentially expressed genes in ΔwblA compared with those of WT (fold change ≥ 2-fold, ≥5-fold and ≥10-fold) at both 24 h and 48 h, which was created using DESeq package, a software platform for identifying differentially expressed genes from RNA-seq data. FC indicates fold change.

RNA-seq analysis revealed a large group of differentially expressed genes, as illustrated in the Heatmap (Figure 2a). Among them, the transcriptional levels of 1162 genes up-regulated 2-fold and 2776 genes down-regulated 2-fold in ΔwblA at 24 h, and 1767 genes up-regulated 2-fold and 2349 genes down-regulated 2-fold in ΔwblA at 48 h. Comparative transcriptome analyses of WT and ΔwblA at both 24 h and 48 h identified 2594 transcripts whose differential expressions were equal or more than 2-fold. Among them, 1433 transcripts were equal or more than 5-fold, and significantly, 1147 transcripts were equal or more than 10-fold (Figure 2b, Table S8). Three blocks were illustrated in the Heatmap (Figure 2a). In block 1, when ΔwblA incubated for 24 h, differentially expressed genes involved in some amino acids and secondary metabolites pathways were sharply decreased compared with those of WT, including the nikkomycin biosynthetic pathway and acarbose, as well as the validamycin biosynthetic pathways (map00525 in KEGG pathway) (Table S9). In block 2, the transcriptional levels of differentially expressed genes increased in the protein biosynthetic pathway, the ribosome biosynthetic pathway (map03010), and the secondary metabolites biosynthetic pathway in ΔwblA at 24 h, such as the TACs biosynthetic pathway, porphyrin and chlorophyll metabolism (map00860). In block 3, when ΔwblA incubated for 48 h, the transcriptional level of differentially expressed genes increased in environmental information processing, signal transducing, quorum sensing pathways and secretion system or transport pathways.

### 2.3. The Silent Nikkomycin Pathway and the Activated Tylosin Analogue Compounds (TACs) Biosynthetic Pathway in ΔwblA

**Figure 3:**
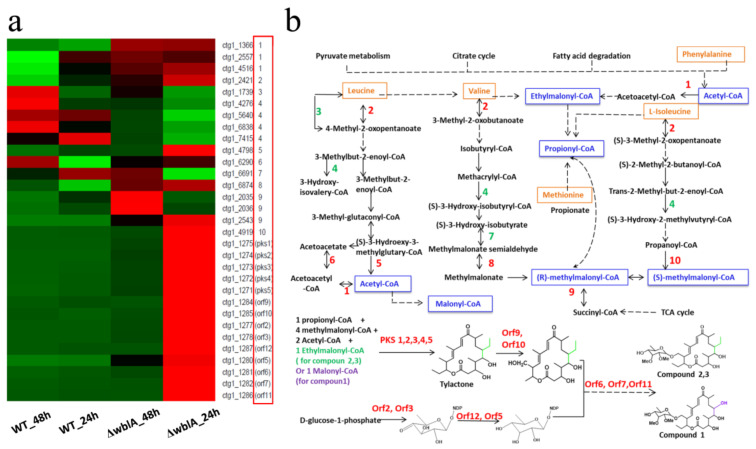
Differentially expressed genes in tylosin analogue compounds (TACs) biosynthesis in WT and ΔwblA. (**a**) Analysis of significantly expressed genes in both WT and ΔwblA at 24 h and 48 h. |log2FC| ≥ 1 and *p*_adjust_ < 0.001. Expression variance for each gene was indicated by different colors: red (up-regulated genes), green (down-regulated genes) and black (|log2FC| < 1). The numbers or gene names in the red box are corresponding to the catalytic reaction steps involved in (**b**). (**b**) Proposed pathway diagram of TACs biosynthesis and the relevant amino acids catabolism. The blue boxes represent the precursors that incorporated into tylactone, and the orange boxes represent the precursors derived from the catabolism of five amino acids. The numbers 1–10 denote differentially expressed genes in 10 reaction steps of deduced KEGG pathway. Green numbers denote down-regulated genes encoding enzymes responsible for these reaction steps, red numbers denote up-regulated genes encoding enzymes responsible for these reaction steps. Dotted arrows represent multiple genes responsible for these reaction steps.

Detailed analysis of the transcriptomics revealed a significant up-regulation of TAC biosynthetic genes (from *pks1* to *orf11*) in ΔwblA (Table 2, Figure 3a). Except for *orf5* which has a 2.8-fold increase, the transcriptional levels of the TAC biosynthetic genes all showed a 14–192-fold increase at 24 h (Table 2, the column of fold change of ΔwblA_24 h against WT_24 h). Moreover, the transcriptional up-regulation of the genes *ctg1_2035* or *ctg1_2036* encoding methylmalonyl-CoA mutase and *ctg1_2557* or *ctg1_4516* encoding acetyl-CoA C-acetyltransferase at 48 h is much higher than that at 24 h; it seems that these genes might be of importance for the biosynthesis of tylosin analogue compounds (TACs). Meanwhile, with the production of the TAC compounds at 48 h, the transcriptional levels of the TACs cluster-situated biosynthetic genes increased approximately 1–10 folds in ΔwblA compared with those of wild-type 7100 at 48 h, even though their transcriptional levels are lower than those at 24 h (Table 2). We speculated that the transcriptional fold change of these genes may be the main factor leading to the production of the the TACs. The decrease of fold change of ΔwblA/WT at 48 h versus fold change of ΔwblA/WT at 24 h may be related to the feedback negative regulation.

The transcription of the genes responsible for the TAC precursor biosynthesis was also analyzed at both 24 h and 48 h. Acetyl-CoA, methylmalonyl-CoA, ethylmalonyl-CoA and malonyl-CoA were proposed to be involved in the tylactone’s assembly (Figure 3b). These precursors or intermediates are derived from primary metabolic pathways as amino acid catabolism, fatty acids catabolism, citrate cycle or pyruvate metabolism. The gene (*ctg1_4798*) responsible for the biosynthesis of acetyl-CoA from leucine metabolism (step 5 in Figure 3b) was transcriptionally up-regulated (6.5-fold increase at 24 h) in ΔwblA. Meanwhile, the genes (*ctg1_4276*, *ctg1_5640*, *ctg1_6838* and *ctg1_7415*) catalyzing the branched metabolic pathway in leucine catabolism (step 4 in Figure 3b) were down-regulated, which was proposed to prompt the intermediates’ flow to the formation of acetyl-CoA. The transcriptional levels of the methylmalonyl-CoA biosynthetic genes including *ctg1_4919* (step 10 in Figure 3b) and ethylmalonyl-CoA biosynthesis related genes (*ctg1_1366*, *ctg1_2557*, *ctg1_4516*, step 1 in Figure 3b) were all apparently increased to supply more precursors for TAC biosynthesis. Transcriptional analysis of the precursors biosynthetic genes will be helpful for improvement of TACs’ production by optimizing the metabolic pathways.

Nikkomycins, a group of peptidyl nucleoside antibiotics, are a potent competitive inhibitor of fungal chitin synthases, since their chemical structure is similar to the natural substrate of chitin synthase, UDPN-acetylglucosamine [31]. The abolishment of nikkomycin production in ΔwblA was also approved by analyzing the transcripts. Nearly all the nikkomycin biosynthetic genes were found to be silent at both 24 h and 48 h in ΔwblA (Figure S2a,b and Table S9). Since no direct binding was observed between WblA and the related promoters, the mechanism that *wblA* disruption activated TACs’ production and simultaneously abolished nikkomycin biosynthesis is still unknown; more investigations need to be performed.

### 2.4. Production of an Anthracycline Antibiotic Oviedomycin Activated by Disrupting Genes san7324 plus san7324L

**Figure 4:**
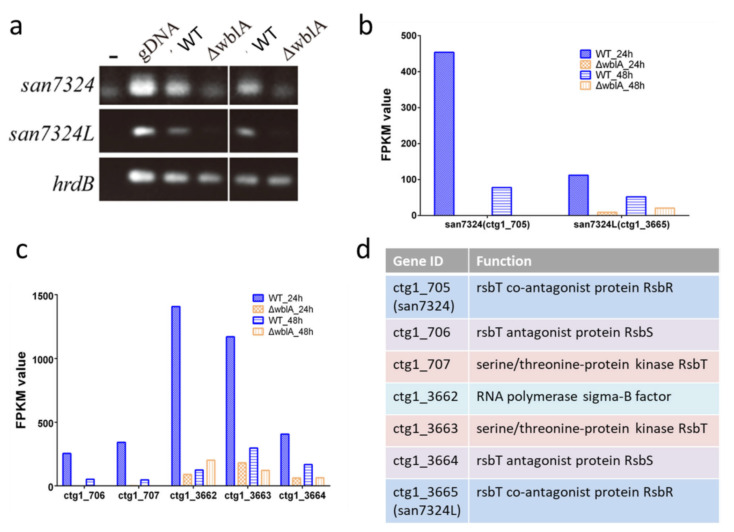
Transcriptional analysis of *san 7324* and *san 7324L* in both *S. ansochromogenes* 7100 and ∆wblA. (**a**) RT-PCR analysis of *san7324* and *san7324L*, -: no DNA template was added. PCR amplifications with cDNA as template were repeated twice. (**b**) FPKM value of *san 7324* and *san7324L* from RNAseq data. (**c**) FPKM value of flanking genes adjacent to *san 7324* and *san 7324L*. (**d**) Gene annotation of ctg1_705–707 and ctg1_3662–3665.

In ΔwblA, transcription of an *rsbR* homologous gene *ctg1_705* (named as *san7324*) in RNA-seq data was found to be dramatically decreased, which was consistent with the RT-PCR results (Figure 4a,b), implying that it was positively regulated by WblA. RsbR encoded by *rsbR* is a positive regulator modulating sigma factor B activity in *Bacillus subtilis* [32,33,34]. The disruption of *san7324* in *S. ansochromogenes* 7100 abolished nikkomycin production (Figure S3). However, unlike that in ΔwblA*,* the production of tylosin analogues in Δsan7324 was not detected [35]. One hypothesis is that a *san7324* homologous gene *ctg1_3665* (named as *san7324L*), which was almost not transcribed in ΔwblA, might play the same function, while *san7324* was deficient. In addition, their flanking genes (encoding RsbS, RsbT or sigma factor) also showed low transcriptional levels (Figure 4c,d).

**Figure 5:**
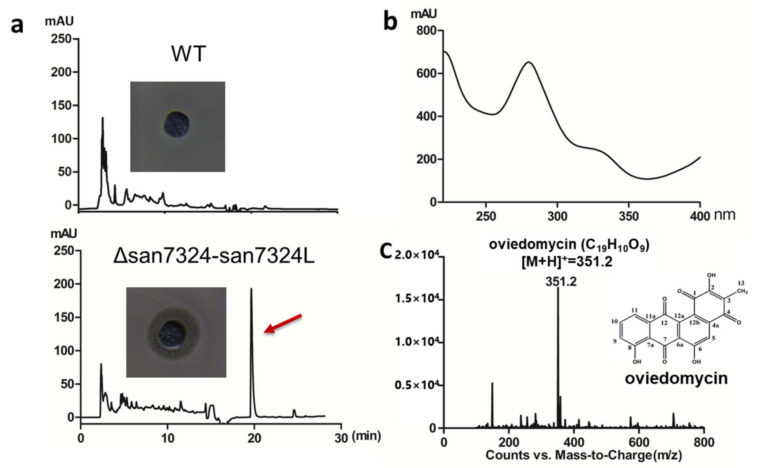
Identification of oviedomycin in *S. ansochromogenes* 7100 (WT) and Δsan7324–7324L. (**a**) HPLC analysis of fermentation supernatant of WT and Δsan7324–7324L. (**b**) UV absorption spectrum of oviedomycin. (**c**) Oviedomycin [M + H]^+^ signal at *m*/*z* 351.2 was analyzed by MS and the structure was shown.

Thus, the double mutation of *san7324* and *san7324L* (Δsan7324/7324L) was constructed and the HPLC results showed TACs were still not be observed. To our surprise, a new peak with maximum absorbance at 280 nm was detected. This compound exhibited an obvious inhibition zone against *Bacillus subtilis* (Figure 5a,b). Mass spectrometry (MS) analysis showed a [M + H]^+^ signal at *m*/*z* 351.2, which is consistent with oviedomycin (Figure 5c). Oviedomycin is an anthracycline antibiotic which had been previously activated in ΔadpA [36]. The production of oviedomycin implied that RsbR homologs might negatively regulate oviedomycin biosynthesis.

### 2.5. Understanding on Activation of Cryptic Antibiotic Biosynthetic Gene Clusters Guided by RNA-seq Data

The TACs biosynthesis was activated by the disruption of *wblA*, but the mechanism of the action of WblA is quite complicated because WblA as a global regulator influences a variety of target genes. Furthermore, there is a serious challenge in obtaining sufficient and active WblA protein (an unstable protein) to perform the protein-DNA interaction experiment. However, the RNA-seq data indicated that some sets of genes involved in several precursors’ (Acetyl-CoA, methylmalonyl-CoA etc.) biosynthesis up-regulated in ΔwblA, which may be helpful for the biosynthesis of TACs. In addition to focusing on the role of WblA in secondary metabolites biosynthesis and morphological differentiation, we also noticed that the transcription of *wblA* (*ctg1_3506*) itself maintained at a high level in its parent strain, but decreased to 0.35-fold and 0.1-fold in ΔwblA at 24 h and 48 h, respectively. Thus, WblA seems to be a positive regulator for its own transcription, and the detailed mechanism of its action remains to be revealed.

Prior to making a decision on target genes for other products’ biosynthesis, we screened the differentially expressed genes in ΔwblA and found the transcription of *san7324* and *san7324L* was substantially down-regulated more than 10-fold in ΔwblA at 24 h, and also their deduced products (enzymes) are probably involved in biosynthetic pathways, therefore the two genes were selected for further experiment. The results demonstrated that oviedomycin production can be triggered by the disruption of *san7324* and *san7324L,* but further investigations on their specific roles need to be conducted in the future. The function of Wbl family proteins has been reported in a number of studies. Some of them had the capacity to form a protein-protein complex and change the structural conformation to regulate its targets, while some Wbl proteins can also modulate sigma factors [24,25,37,38], suggesting that they may play broad roles in multiple pathways. In conclusion, mining of the RNA-seq data provided a useful alternative approach for searching target genes, which may be related to secondary metabolisms. It would be more effective to activate silent gene clusters if combined with other strategies.

## 3. Materials and Methods

### 3.1. Strains and Growth Condition

*Streptomyces ansochromogenes* 7100, a nikkomycin producer, was used as the wild-type strain. *S. ansochromogenes* and its derivatives were grown on MS (2% soya flour, 2% mannitol) agar at 28 °C for harvesting spores. For nikkomycin or tylosin analogue compounds (1, 2, 3) production, the strains were firstly grown in SP (3% mannitol, 1% potato starch, 0.8% yeast extract, 0.5% neutral soy peptone) as a seed medium and then inoculated 5% (*v*/*v* = 5/100) seed culture into SP (3% mannitol, 1% potato starch, 0.8% yeast extract, 0.5% neutral soy peptone) for further fermentation at 28 °C for 5–8 days. For the production of oviedomycin, *S. ansochromogenes* 7100 and its derivatives were grown in the same seed medium and then 1% (*v*/*v* = 1/100) of seed culture was inoculated into a GYM medium (0.4% glucose, 0.4% malt extract, 1% yeast extract) for further fermentation at 28 °C for 6 days. Disruption mutants of *san 7324 (ctg1_705)*, *san7324L (ctg1_3665)*, the double gene disruption mutant (Δsan7324–7324L) and complementary strain were previously constructed [35]. The *wblA* disruption mutant (ΔwblA) and its complementary strain were previously constructed [28].

### 3.2. HPLC Analysis of Oviedomycin

The cultures were filtered through gauze, extracted with chloroform, dried in vacuo and then re-dissolved in 1 mL of methanol. The detection of oviedomycin was performed by HPLC with a ZORBAX SB-C18 reverse phase column (4.6 mm × 250 mm, 5 μm; Agilent) as described previously [36].

### 3.3. PacBio Sequencing, Assembly and Gene Annotation

The 64 μg of high-quality genomic DNA was used to prepare the SMRTbell library, and then sequenced on a PacBio RS II platform [39].

Prior to assembly, raw reads containing low-quality were filtered. After pre-process, a total of 2.09 Gb of the usable data were retained for the following assembly (Table S1). We used Canu software to assemble the subreads into scaffolds. The final assembly size is 9.56 Mb with scaffold N50 of 9.21M (Table S2). Repeat sequences were identified by Repeat Masker software. The estimated repeat sequences account for 2.37% of the genome assemblies (Table S3). Prodigal was performed to predict genes. In total, 8325 protein-encoding genes were generated for *S. ansochromogenes* 7100 (Table S4). The tRNA-coding genes were predicted by tRNAscan-SE (v1.3.1) software. rRNA and other ncRNA-coding genes were identified by Infernal 1.1 against Rfam database (http://rfam.xfam.org/, access on 9 September 2021) (Table S5). Annotation of predicted genes was conducted by BLASTP against NCBI non-redundant protein sequence database (NR), Swissprot and TrEMBL protein databases with *E*-value < 10^−5^. Gene Ontology (GO) terms were retrieved from NR outputs by Blast2GO software [40]. Pathway analysis was performed using the Kyoto Encyclopedia of Genes and Genomes (KEGG) annotation service KAAS (Table S6).

### 3.4. RNA Isolation, Library Construction and Sequencing 

The mycelia of S. *ansochromogenes* 7100 and ΔwblA were harvested at 24 h and 48 h from a 50-mL culture by centrifugation at 12,000 rpm for 15 min at 4 °C. The cell pellets were quickly frozen in liquid N_2_ and then stored at −80 °C. Total RNAs were isolated using the TRIzol reagent, and DNase I was used to digest the total DNA according to the manufacturer’s protocol. RNA degradation or contamination was monitored with 1% agarose gel electrophoresis. RNA purity was checked by using the NanoPhotometer^®^ spectrophotometer (IMPLEN, Westlake Village, CA, USA). Strand-specific RNA-Seq libraries were constructed and sequenced by the Illumina HiSeq 2000 system, 100 bp paired-end reads were generated and the RNA-seq data were analyzed by Majorbio company.

### 3.5. Quality Control and Reads Mapping to the Reference Genome

The raw data were filtered using a Perl program by removing low-quality sequences, reads with more than 5% of N bases (unknown bases) and reads containing adaptor sequences. Then the clean data were mapped to the genome sequences of *S. ansochromogenes* 7100 by the Bowtie2 program [41].

### 3.6. Quantification of Gene Expression Level and Differential Expression Analysis

An abundance of transcripts was estimated using RSEM and transformed into formula FPKM (fragments per kilobase per million mapped reads). Differentially expressed genes (DEGs) were calculated by using R package DESeq [42]. Genes with an adjusted *p*-value (*p*-adjust) < 0.001 found by DESeq were assigned as differential expressions, and those with fold change |Log2FC| ≥ 1 were defined to be either up- or down-regulated genes, respectively. The Heatmap was made by R package heatmap.2.

### 3.7. RT-PCR Analysis

For RT-PCR, 1 μg of RNA was reverse-transcribed to cDNA using a SuperRT cDNA Synthesis Kit (CWBIO, Beijing, China) [43]. To perform transcriptional analysis of *san7324* and *san7324L*, primer pair (san7324-RT-F: ACGCTGCGGCTGGTGGTCAT, san7324-RT-R: CCGTTCCCTCCCACAGCTTGAT) and another primer pair (san7324L-RT-F: CCCGTCATCAAGCTGTGGGAGG, san7324L-RT-R: TCAGGTGCTGGGCCACGAAC) were respectively used for PCR amplification with cDNAs of WT and ΔwblA as templates. The transcription of *hrdB* using primers (hrdB-RT-F: GCTGGCCAAGGAACTCGACAT, hrdB-RT-R: CGAAGCGCATGGAGACGACG) was used as an internal control.

### 3.8. Data Availability

The genomic DNA sequence of *S. ansochromogenes* 7100 is deposited in the China National Microbiology Data Center (NMDC) with accession numbers NMDC60029072. RNA-seq raw data were deposited in the China National Microbiology Data Center (NMDC) with accession numbers NMDC40009909 (WT_24 h), NMDC40009910 (WT_48 h), NMDC40009911 (ΔwblA_24 h), NMDC40009912 (ΔwblA_48 h).

## 4. Conclusions

RNA-seq analyses demonstrated that high-level transcription of most genes was associated with the production of nikkomycin in WT and TACs in ΔwblA appeared at 24 h, but decreased significantly at 48 h. Interestingly, double mutation of *san7324* and *san7324L* (Δsan7324/7324L) led to the production of oviedomycin, which was not produced in wild-type *S. ansochromogenes* under laboratory conditions. It was suggested that RsbR homologs (San7324/7324L) might negatively regulate oviedomycin biosynthesis. Moreover, the findings would provide an effective approach to dissect the production and biosynthetic pathways of natural secondary metabolites, and also provide insights into the activation of cryptic natural products.

## Figures and Tables

**Table 1:** AntiSMASH analysis of genome sequence of the *S. ansochromogenes* 7100.

Region	Type	From (bp)	To (bp)	Most Similar Known Cluster	Similarity
Region 1	NRPS, NRPS-like, betalactone, nucleoside	118,096	202,793	neopolyoxin C	100%
Region 2	lanthipeptide-class-ii, terpene	287,202	318,846	heronamide A/heronamide B/heronamide C/heronamide D/heronamide E/heronamide F	8%
Region 3	NRPS, T1PKS, terpene	413,811	476,353	eponemycin	21%
Region 4	ectoine	579,945	589,601	ectoine	75%
Region 5	NRPS-like	681,454	723,406	livipeptin	100%
Region 6	terpene, T1PKS, PKS-like, NRPS, RiPP-like	941,834	1,090,502	filipin	38%
Region 7	RiPP-like	1,402,262	1,411,931		-
Region 8	T2PKS, T1PKS	1,481,390	1,632,620	spiramycin	46%
Region 9	terpene	1,658,532	1,682,854	hopene	92%
Region 10	butyrolactone	2,066,935	2,076,146	lactonamycin	5%
Region 11	siderophore	2,110,497	2,123,136	-	-
Region 12	terpene	2,269,830	2,290,914	ashimides	16%
Region 13	butyrolactone	2,300,561	2,311,180	prejadomycin/rabelomycin/gaudimycin C/gaudimycin D/UWM6/ gaudimycin A	6%
Region 14	RiPP-like	2,317,869	2,327,714	streptonigrin	5%
Region 15	butyrolactone	2,408,057	2,418,502	-	-
Region 16	NRPS, NAPAA	2,440,769	2,482,644	stenothricin	13%
Region 17	siderophore	2,636,536	2,648,477	-	-
Region 18	thioamitides, RiPP-like	3,052,710	3,074,720	A-503083 A/A-503083 B/A-503083 E/A-503083 F	3%
Region 19	terpene	3,258,980	3,279,405	albaflavenone	100%
Region 20	NRPS, RRE-containing	3,416,101	3,487,684	granaticin	8%
Region 21	T2PKS	4,177,007	4,248,593	oviedomycin	95%
Region 22	betalactone	4,526,013	4,553,501	divergolide A/divergolide B/divergolide C/divergolide D	6%
Region 23	terpene	5,147,528	5,167,076	mitomycin	3%
Region 24	siderophore	5,623,772	5,635,544	desferrioxamin B/desferrioxamine E	83%
Region 25	melanin, RRE-containing, phosphonate	5,736,851	5,791,306	fosfazinomycin A	78%
Region 26	ectoine	6,302,336	6,311,857	showdomycin	52%
Region 27	T2PKS	6,332,547	6,405,060	spore pigment	83%
Region 28	terpene	6,423,141	6,443,139	neocarazostatin A	100%
Region 29	ectoine	6,762,458	6,772,862	ectoine	100%
Region 30	NRPS-like, NRPS, terpene	7,351,300	7,421,452	enduracidin	4%
Region 31	T3PKS	7,458,394	7,497,631	herboxidiene	7%
Region 32	T1PKS, NRPS, PKS-like	7,669,936	7,736,833	LL-D49194α1 (LLD)	3%
Region 33	melanin	7,963,719	7,974,099	melanin	57%
Region 34	T1PKS	8,145,007	8,259,932	sceliphrolactam	92%
Region 35	T3PKS, lanthipeptide-class-i, T1PKS	8,397,854	8,505,277	A-47934	8%
Region 36	lanthipeptide-class-i	8,764,849	8,790,087	primycin	8%
Region 37	butyrolactone	8,824,305	8,835,291		-
Region 38	lassopeptide	8,842,116	8,864,247	SSV-2083	36%
Region 39	NRPS, lassopeptide	8,883,560	8,950,374	achromosin	100%
Region 40	terpene	9,046,490	9,072,076	carotenoid	63%
Region 41	lassopeptide	9,413,607	9,436,052	-	-

Genomic sequence of the *S. ansochromogenes* 7100 was submitted to the antiSMASH program [30]. A total of 41 gene clusters were identified. Among them, gene clusters in region 1 and region 8 were responsible for the biosynthesis of nikkomycin and tylosin analogue compounds (TAC1,2,3), respectively.

**Table 2:** Differentially expressed genes in the biosynthesis of tylosin analogue compounds (TACs).

Gene ID	Step or Gene Name	KO_id in KEGG Pathway	Fold Change (ΔwblA_24 h/WT_24 h)	Fold Change (ΔwblA_48 h/WT_48 h)	Start	End	Strand	Function	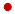 Up 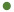 Down
ctg1_1366	step 1	K00626	2.9	2.5	1701042	1702256	−	acetyl-CoA C-acetyltransferase	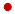
ctg1_2557	step 1	K00626	1.0	8.3	3127039	3128238	−	acetyl-CoA C-acetyltransferase	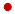
ctg1_4516	step 1	K00626	1.6	7.3	5304370	5305590	−	acetyl-CoA C-acetyltransferase	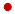
ctg1_2421	step 2	K00826	1.7	2.6	2966975	2968063	−	branched-chain amino acid aminotransferase	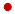
ctg1_1739	step 3	K00263	0.6	0.6	2144241	2145296	+	leucine dehydrogenase	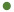
ctg1_4276	step 4	K01692	0.1	0.2	5031893	5032639	+	enoyl-CoA hydratase	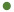
ctg1_5640	step 4	K01692	0.2	0.5	6578023	6578784	−	enoyl-CoA hydratase	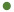
ctg1_6838	step 4	K01692	0.1	0.3	7905240	7906040	+	enoyl-CoA hydratase	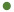
ctg1_7415	step 4	K01692	0.2	0.7	8560092	8560883	+	enoyl-CoA hydratase	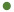
ctg1_4798	step 5	K01640	6.5	3.9	5638643	5639584	−	hydroxymethylglutaryl-CoA lyase	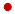
ctg1_6290	step 6	K01907	3.2	0.7	7305337	7307313	+	acetoacetyl-CoA synthetase	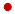
ctg1_6691	step 7	K00020	0.4	1.3	7743630	7744517	−	3-hydroxyisobutyrate dehydrogenase	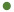
ctg1_6874	step 8	K00128	7.7	2.1	7947480	7948802	+	aldehyde dehydrogenase (NAD+)	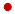
ctg1_2035	step 9	K01847	0.6	14.6	2497253	2499067	+	methylmalonyl-CoA mutase	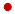
ctg1_2036	step 9	K01847	1.0	12.8	2499067	2501241	+	methylmalonyl-CoA mutase	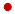
ctg1_2543	step 9	K01848	2.8	1.7	3114436	3116136	−	methylmalonyl-CoA mutase, N-terminal domain	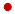
ctg1_4919	step 10	K01965	15.9	2.0	5767295	5768551	−	propionyl-CoA carboxylase alpha chain	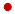
ctg1_1275	PKS1	*	90.0	7.0	1574987	1588348	−	Polyketone synthase	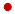
ctg1_1274	PKS2	*	27.4	3.7	1568736	1574990	−	Polyketone synthase	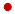
ctg1_1273	PKS3	*	32.9	5.0	1557006	1568672	−	Polyketone synthase	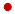
ctg1_1272	PKS4	*	37.7	8.4	1552212	1556942	−	Polyketone synthase	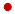
ctg1_1271	PKS5	*	20.3	4.2	1545995	1552108	−	Polyketone synthase	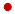
ctg1_1284	Orf9	*	14.4	1.7	1599380	1599619	−	ferredoxin	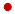
ctg1_1285	Orf10	*	25.7	1.5	1599659	1600816	−	cytochrome p450	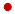
ctg1_1277	Orf2	*	72.2	5.3	1590321	1591208	+	dTDP-glucose_synthase	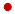
ctg1_1278	Orf3	*	192.0	11.3	1591278	1592276	+	dTDP-glucose 4,6-dehydratase	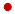
ctg1_1287	Orf12	*	39.2	4.3	1601980	1602597	+	dTDP-4-dehydrorhamnose 3,5-epimerase	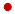
ctg1_1280	Orf5	*	2.8	1.6	1594851	1595702	+	4-ketoreductase_in_D-allose_pathway	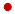
ctg1_1281	Orf6	*	22.3	4.1	1595710	1597002	−	D-allose_glycosyltransferase	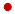
ctg1_1282	Orf7	*	40.3	3.8	1597061	1598248	−	2’OH-methyltransferase	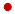
ctg1_1286	Orf11	*	40.5	3.5	1601199	1601969	+	methyltransferase	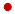

* indicates the cluster-situated structural genes, fold change denotes the gene FPKM value ratio of ΔwblA against WT at 24 h or 48 h.

## Data Availability

The data presented in this study are openly available in China National Microbiology Data Center (NMDC), reference number of the BioProject is [NMDC10017856]. Data of genomic DNA of *S. ansochromogenes* 7100 can be found in NMDC60029072, RNA-seq data of *S. ansochromogenes* 7100 can be found in NMDC40009909 (WT_24 h), NMDC40009910 (WT_48 h), NMDC40009911 (ΔwblA_24 h), NMDC40009912 (ΔwblA_48 h).

## Supplementary Materials

The following are available online at https://www.mdpi.com/article/10.3390/antibiotics10091097/s1, Figure S1: Saturation curves of sequencing data in RNA-seq, Figure S2: Differentially expressed genes involved in nikkomycin biosynthesis in both WT and ΔwblA, Figure S3: HPLC analysis of nikkomycin production in WT and Δsan7324, Table S1: Statistics of filtered sequencing data, Table S2: Assembly of genome sequencing data, Table S3: Summary of repeat elements identified, Table S4: Statistics of prediction of the proteins, Table S5: Non-coding RNAs, Table S6: Annotation of genes, Table S7: Genome mapping statistics of RNA-seq data, Table S8: Significantly expressed genes in Venn diagram, Table S9: FPKM value of part of differentially expressed genes.
