# Supplementary material for: Activation of Cryptic Antibiotic Biosynthetic Gene Clusters Guided by RNA-seq Data from Both Streptomyces ansochromogenes and ΔwblA
Source: Antibiotics (Basel). 2021 Sep 10;10(9):1097. doi: 10.3390/antibiotics10091097 (PMC8465540; doi:10.3390/antibiotics10091097)
Supplement: Supplementary file 1 [file antibiotics-10-01097-s001.zip › Supplementary Materials/Proof--Supplemental Figures.pdf]

Supplementary Materials
Figures

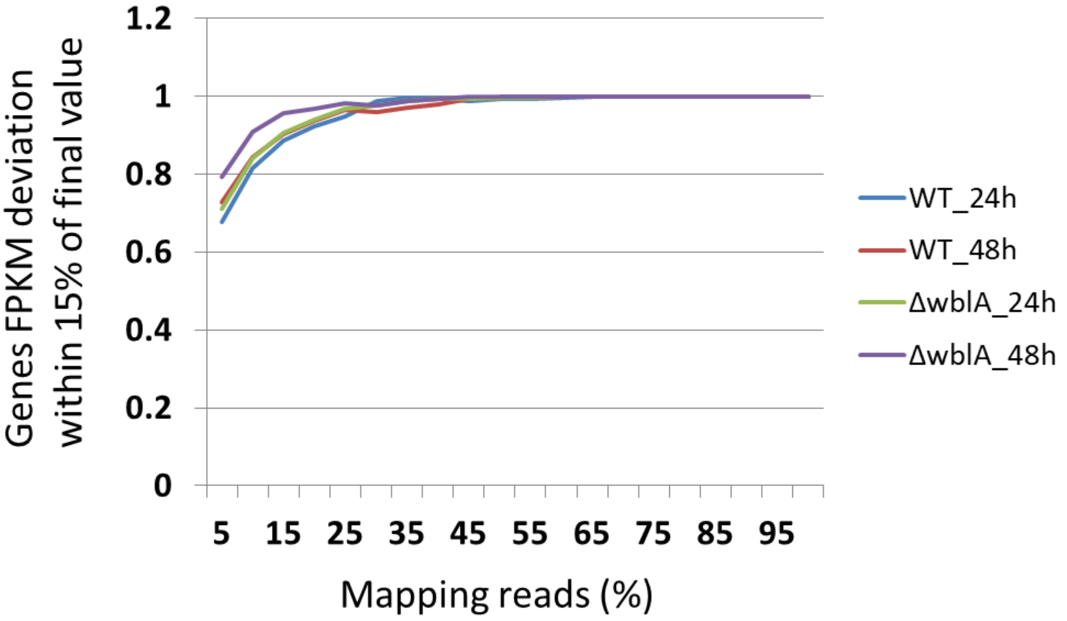

Figure S1. Saturation curves of sequencing data in RNA-seq. The X axis is the percentage of effective reads comparison (e.g., 80 means that 80% of the comparison sequencing data were randomly selected to calculate the expression level of this gene). The Y axis is the deviation ratio (error within 15%) between the expression level and the final value under the sampling condition (e.g., 0.8 means that 80% of the gene expression level has an error within 15%). The value is closer to 1, the expression level is more saturated . Each color line represents the saturation curve of gene expression at different expression levels in the eight samples.

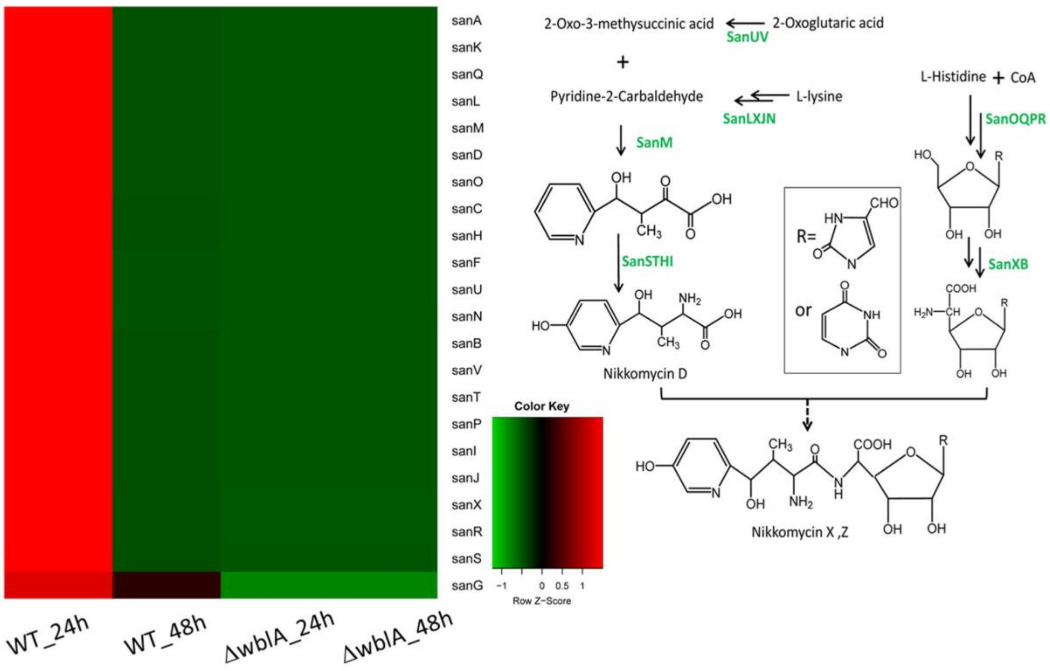

Figure S2. Differentially expressed genes involved in nikkomycin biosynthesis in both WT and ΔwblA. (a) Heatmap represents the transcriptional level of nikkomycin biosynthetic genes in both WT and ΔwblA. **(b)** Diagram of nikkomycin X and Z biosynthesis. Double arrows represent multiple reaction steps, and dashed arrows represent unknown paths.

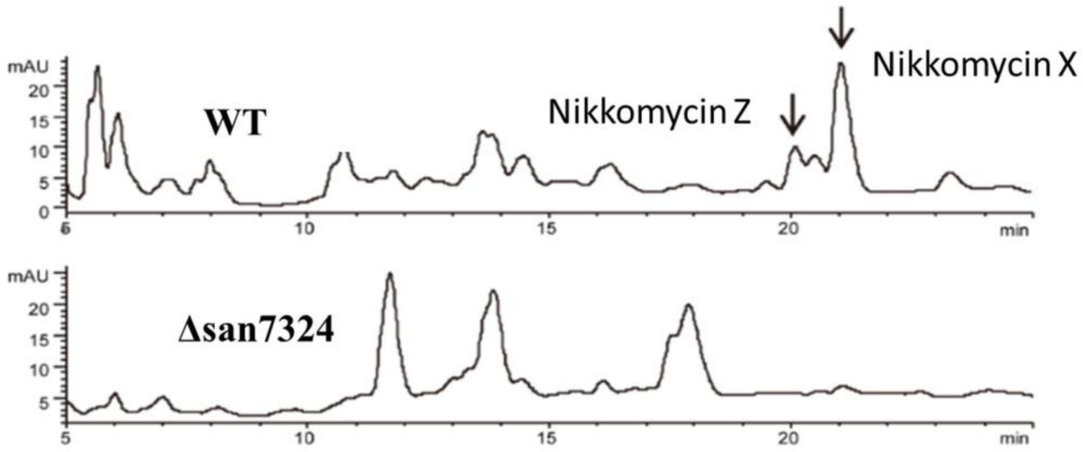

Figure S3. HPLC analysis of nikkomycin production in WT and Δsan7324. San7324 （Ctg1_705）encoding by *san7324* is a RsbR homolog.
